# *The 677C>T* (rs1801133) Polymorphism in the *MTHFR* Gene Contributes to Colorectal Cancer Risk: A Meta-Analysis Based on 71 Research Studies

**DOI:** 10.1371/journal.pone.0055332

**Published:** 2013-02-20

**Authors:** Zan Teng, Lei Wang, Shuang Cai, Ping Yu, Jin Wang, Jing Gong, Yunpeng Liu

**Affiliations:** 1 Department of Medical Oncology, the First Hospital of China Medical University, Shenyang, Liaoning, P.R. China; 2 Department of Geriatrics, the First Hospital of China Medical University, Shenyang, Liaoning, P.R. China; 3 Department of Pharmacy, the First Hospital of China Medical University, Shenyang, Liaoning, P.R. China; Ospedale Pediatrico Bambino Gesù, Italy

## Abstract

**Background:**

The 677C>T polymorphism of methylenetetrahydrofolate reductase *(MTHFR)* gene is considered to have a significant effect on colorectal cancer susceptibility, but the results are inconsistent. In order to investigate the association between the *MTHFR* 677C>T polymorphism and the risk of colorectal cancer, a meta-analysis was held based on 71 published studies.

**Methods:**

Eligible studies were identified through searching the MEDLINE, EMBASE, PubMed, Web of Science, Chinese Biomedical Literature database (CBM) and CNKI database. Odds ratios (OR) and 95% confidence intervals (CIs) were used to assess the association. The statistical heterogeneity across studies was examined with x^2^-based Q-test. Begg's and Egger's test were also carried out to evaluate publication bias. Sensitive and subgroup analysis were also held in this meta-analysis.

**Results:**

Overall, 71 publications including 31,572 cases and 44,066 controls were identified. The *MTHFR* 677 C>T variant genotypes are significantly associated with increased risk of colorectal cancer. In the stratified analysis by ethnicity, significantly increased risks were also found among Caucasians for *CC vs TT* (OR = 1.076; 95%CI =  1.008–1.150; *I^2^ = *52.3%), *CT vs TT* (OR = 1.102; 95%CI = 1.032–1.177; *I^2^ = *51.4%) and dominant model (OR = 1.086; 95%CI = 1.021–1.156; *I^2^ = *53.6%). Asians for *CC vs TT* (OR  = 1.226; 95%CI  = 1.116–1.346; *I^2^  = *55.3%), *CT vs TT* (OR  = 1.180; 95%CI  = 1.079–1.291; *I^2^  = *36.2%), recessive (OR  = 1.069; 95%CI  = 1.003-1.140; *I^2^  = *30.9%) and dominant model (OR  = 1.198; 95%CI  = 1.101-1.303; *I^2^  = *52.4%), and Mixed populations for *CT vs TT* (OR  = 1.142; 95%CI  = 1.005-1.296; *I^2^  = *0.0%). However, no associations were found in Africans for all genetic models.

**Conclusion:**

This meta-analysis suggests that the *MTHFR 677C>T* polymorphism increases the risk for developing colorectal cancer, while there is no association among Africans found in subgroup analysis by ethnicity.

## Introduction

Colorectal cancer (CRC) is a worldwide public health problem,which is the third most commonly diagnosed cancer in males and the second in females with over 1.2 million new CRC patients and 608,700 deaths occurred in the world [1], [2]. Previous studies demonstrated that colorectal carcinogenesis is a complicated multi-step progress involving changes of many oncogenes and tumor suppressor genes induced by the interaction of many factors. Simultaneously, other factors such as alcohol, low methionine, low folate diets, heavy drink, smoking and environmental carcinogenic agents are assumed the possible risk factors. Whereas, not all individuals exposed to the above exogenous risk factors will develop CRC, which suggested that individual susceptibility factors may play an important role in the cancer development.

Methylenetetrahydrofolate reductase (MTHFR) located on chromosome 1 at 1p36.3, is an important enzymes involved in the folate metabolic pathway. It reduces 5,10-methylenetetra-hydrofolate which protects cells from DNA damage induced by uridylate misincorporation to 5-methyltetrahydrofolate, a carbon donor for the homocysteine/methionine conversion. In addition, 5-methyltetrahydrofolate is the major circulating form of folate and is important for DNA synthesis, repair, and methylation [3], [4]. Folate, in its 5- methyl form, participates in single-carbon transfers that occur as part of the synthesis of nucleotides, the synthesis of *S*-adenosyl-methionine, the remethylation of homocysteine to methionine, the methylation of DNA, proteins, neurotransmitters and phospholipids. DNA aberrant methylation might also increase the risk of CRC [5]. It has been considered as one of the molecular mechanisms by which gene expression is regulated [6].

Reduced activity of the MTHFR, due to C677T polymorphisms, increases the pool of 5,10-methy-lene-THF that reduces misincorporation of uracil into DNA, which might lead to double-strand breaks during uracil excision repair processes, and increase chromosomal aberrations risk [7]. C677T (rs1801133) is a single nucleotide polymorphism (SNP) in exon 4 at the folate binding site of the MTHFR gene which influences the general balance between DNA synthesis, repair, and methylation processes. Individuals with the MTHFR 677TT genotype have been shown to have only 30% of the in vitro MTHFR enzyme activity compared with the wild type, whereas those with the heterozygous CT genotype have been found to have 60% of wild-type MTHFR enzyme activity [8]. Up to 15% of individuals are homozygous 677TT for the variant, which is associated with higher plasma homocysteine levels and reduced plasma folate levels [9]. Considering the functional effects of the polymorphisms of these enzymes, it is expected that these gene polymorphisms may be associated with the CRC risk.

Previous epidemiological studies have been conducted trying to clarify the association between the MTHFR C677T polymorphism and CRC susceptibility, but obtain controversial results. To date, we performed a meta-analysis from all eligible studies and provided more accurate estimate the association between the MTHFR C677T polymorphism and the risk of CRC.

## Materials and Methods

### Literature and search strategy

We considered all studies that examined the association of the MFTHR 677C>T polymorphism with CRC. The sources included MEDLINE, EMBASE, PubMed, Web of Science, Chinese Biomedical Literature database (CBM) and CNKI database (last search was update on July 2012). The search strategy to identify all possible studies involved uses the combinations of the following key words: “methylenetetrahydrofolate reductase”, “*MTHFR*”, “*MTHFR C677T*”, “*SNPs*”, “*rs1801133*”; “polymorphism”, “genotype”; “colorectal”, “colon”, “rectal”; “cancer”, “carcinoma”, “adenocarcinoma”. No language restrictions were imposed. The reference lists of review articles, clinical trials, and meta-analyses, were also hand-searched for the collection of other relevant studies, and two authors conducted all searches independently. Non-familial case-control studies were eligible if they determined the distribution for this polymorphism in unrelated patients with CRC and in a concurrent control group of healthy subjects using molecular methods for genotyping. We did not include abstracts or unpublished reports. When overlapping data of the same patient population were included in more than one publication, only the most recent or complete study was used in this meta-analysis.

### Eligibility criteria

The studies accepted for this meta-analysis had to meet the following criteria: (1) utilized platinum-based regimens for patients with pathologically proven colorectal cancer; (2) controls were consisted with normal persons; (3) only cohort studies and case-control studies included in this meta-analysis; (4)evaluation of the *MTHFR C677T* polymorphisms and colorectal cancer risks; (5) The paper should clearly describe the sources of cases and controls; (6) The authors must offer the size of the sample, OR and their 95% CI or the information that can help infer the results in the papers.

Accordingly, major reasons for exclusion of studies were: (1) not designed as case/control or cohort studies; (2) reviews and repeated research studies; (3) not offering the sources of cases and controls and other essential information; (4) control population including malignant tumor patients; (5) duplicated publications.

### Quality assessment

The Newcastle – Ottawa Quality Assessment Scale for cohort studies was used to assess the quality of the studies [10], [11]. This scale is composed of eight items that assess patient selection, study comparability and outcome. The scale was recommended by the Cochrane Non-Randomized Studies Methods Working Group [12]. Two investigators performed quality assessment independently. Disagreement was resolved by consensus.

### Data extraction

Information was carefully extracted from all eligible publications independently by two authors according to the inclusion and exclusion criteria listed above. Disagreement was resolved by discussion between the two authors. The following data were collected from each study: first author's surname, year of publication, ethnicity, and numbers of cases and controls with the *CC*, *CT* and *TT* genotypes, and Hardy – Weinberg equilibrium (*HWE*) in controls, respectively. Different ethnicity descents were categorized as Caucasian, Asian, African, and Mixed population. When studies included subjects of more than one ethnicity and were able to separate, data were extracted separately for each ethnic group. We did not define any minimum number of patients to include a study in our meta-analysis.

### Statistical analysis

We used the crude odds ratios (ORs) with 95% confidence intervals (*CIs*) to assess the strength of this association according to the method of Woolf [13]. Subgroup analyses were done by ethnicity. Both fixed-effects model using the Mantel – Haenszel method [14] and random-effects model using the DerSimonian and Laird method [15] were used to pool the results. The fixed-effects model was used when there was no heterogeneity of the results of studies; otherwise, the random-effects model was used.

Heterogeneity assumption was checked by the Chi-square-based Q-test [16]. A P-value greater than 0.10 for the Q-test indicates a lack of heterogeneity among studies. Simultaneously, it was also assessed using the *I^2^* statistic, which takes values between 0% and 100% with higher values denoting greater degree of heterogeneity (*I^2^* = 0–25%: no heterogeneity; *I^2^* = 25–50%: moderate heterogeneity; *I^2^* = 50–75%: large heterogeneity; *I^2^* = 75–100%: extreme heterogeneity) [17]. The significance of the pooled OR was determined by the Z-test, and P<0.05 was considered as statistically significant. One-way sensitivity analyses were performed to assess the stability of the results. An estimate of potential publication bias was carried out by the funnel plot, Begg's and Egger's test. The significance of the intercept was determined by the t-test suggested by Egger (P<0.05 was considered representative of statistically significant publication bias) [18]. Hardy – Weinberg equilibrium in the control group was tested by the Chi-square test, and P-value of <0.05 was considered significant. All of the calculations were performed using STATA (version 12.0; Stata Corporation, College Station, TX), using two-sided P-values.

## Results

### Study characteristics

**Figure 1:**
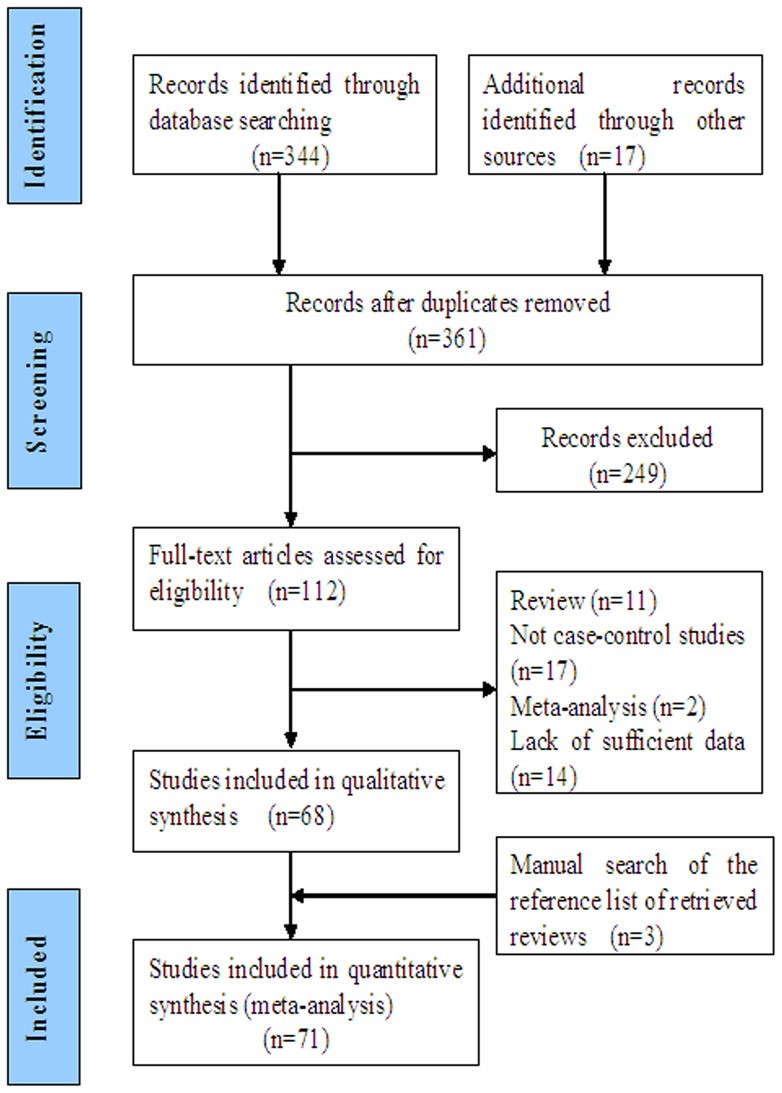
Flow diagram summarizing the search strategy.

Studies relevant to the searching words were retrieved originally. The flow diagram of the literature search strategy is shown in **Figure** **1**. 71 publications involving 31,572 CRC cases and 44,066 controls were ultimately analyzed, addressing the association between *MTHFR C677T* polymorphism and CRC susceptibility was preliminarily eligible [19]–[89]. The main characteristics of these studies included in this meta-analysis were presented in **Table** **S1**. All the cases were histologically confirmed. Controls were mainly healthy populations. Therefore, each study was considered separately for pooling subgroup analysis. There were a total of 71 studies, including 77 independent case-control or cohort researches, which included 38 groups of Caucasians, 27 groups of Asians, 8 groups of Mixed populations and 4 groups of Africans. The distribution of genotypes in the controls of all studies was in agreement with Hardy – Weinberg equilibrium except 13 researches [20], [21], [24], [40], [42], [43], [46], [49], [58], [75], [81], [84], [88].

### Meta-analysis results

**Figure 2:**
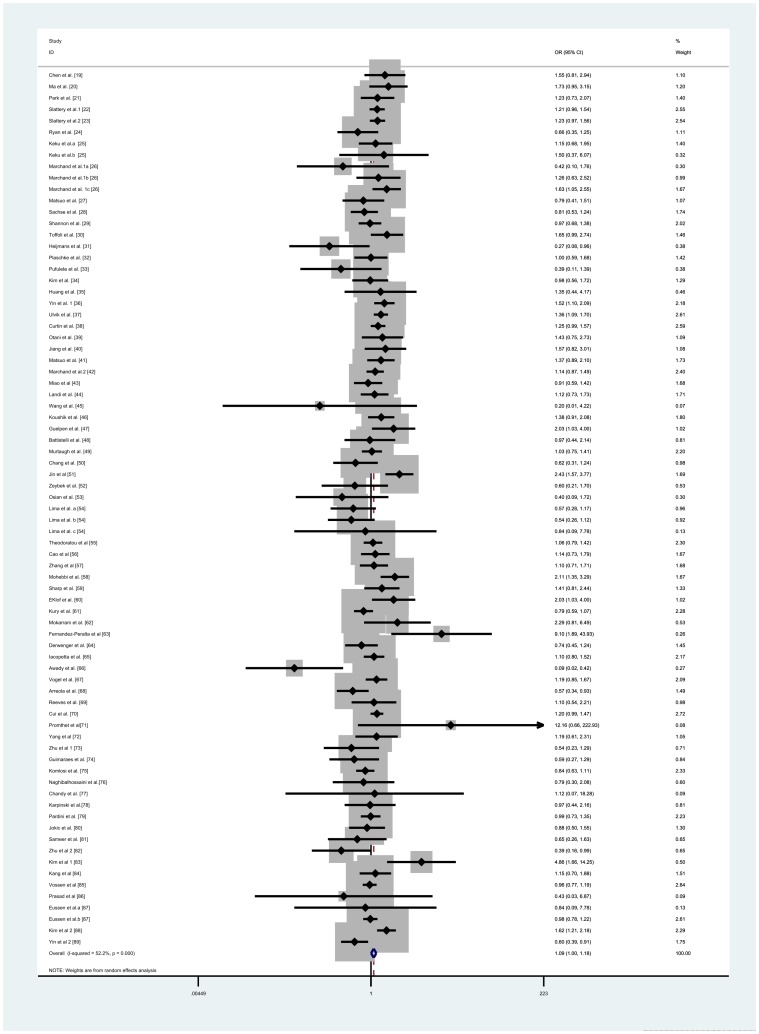
Forest plot of colorectal cancer susceptibility associated with MTHFR 677C>T polymorphism (for *CC vs TT*).

**Figure 3:**
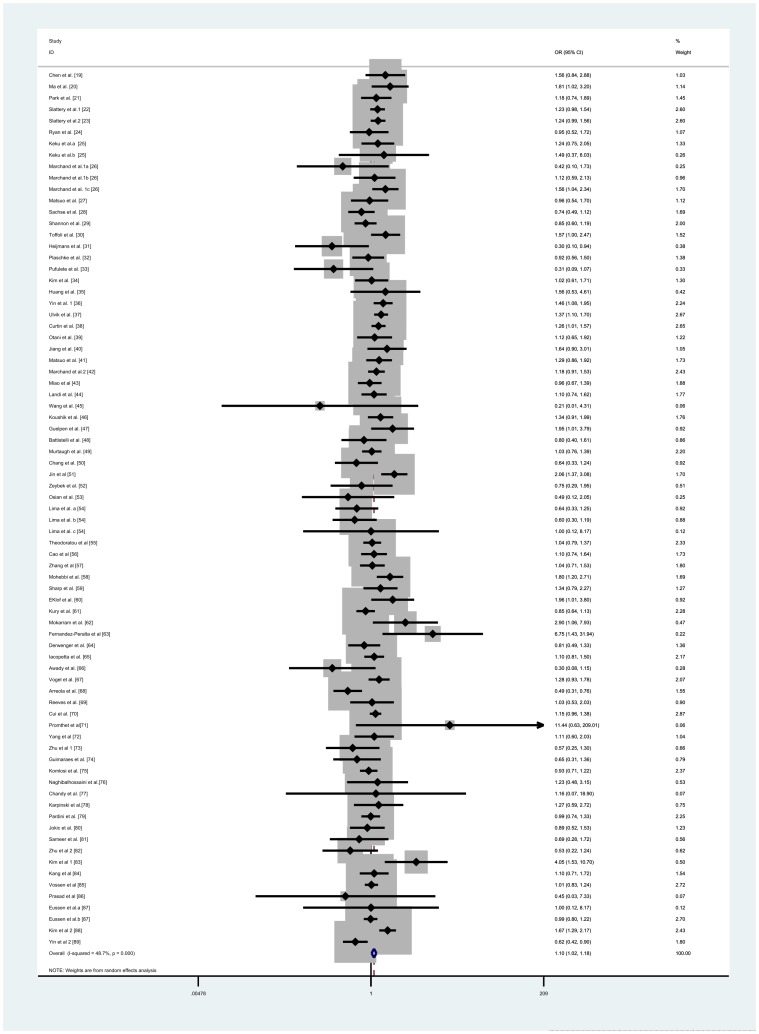
Forest plot of colorectal cancer susceptibility associated with *MTHFR 677C>T* polymorphism at dominant model (*CC + CT* vs *TT*).

The main results of this meta-analysis were listed in **Table** **1**. Overall, significantly elevated CRC risk were associated with some genetic models when all the eligible studies were pooled into the meta-analysis (OR = 1.089; 95%CI = 1.002–1.183; *I^2^* = 52.2% for *CC vs TT*, **Figure** **2**; OR = 1.113; 95%CI = 1.037–1.195; *I^2^*  = 38.5% for *CT vs TT*, **Figure** **S1**; OR = 1.097; 95%CI = 1.019–1.182; *I^2^* = 48.7% for dominant model, **Figure** **3**). In contrast, there were no associations were found in other genetic models. Simultaneously, the C-allele genotype was not associated with an increased CRC risk compared with the T-allele genotype (OR  = 1.014; 95%CI = 0.973–1.056; *I^2^  = *60.9%, **Figure** **S2**).

**Table 1:** Meta-analysis on the association between 677 C>T *MTHFR* polymorphism and colorectal cancer susceptibility.

Variables	Study number	Statistic	Test of heterogeneity			Test of Association	
		Model	Q	*P*	*I^2^*	OR(95% CL)	*P*
**CC vs TT**							
Total	77	Random	159.08	0.000	52.2%	1.089 (1.002 1.183)	**0.044**
Caucasian	38	Random	77.51	0.000	52.3%	1.076(1.008 1.150)	**0.029**
Asian	27	Random	58.18	0.000	55.3%	1.226(1.116 1.346)	**0.000**
Mixed population	8	Fixed	7.70	0.360	9.0%	1.112(0.980 1.262)	0.098
African	4	Fixed	7.61	0.055	60.6%	1.119(1.065 1.176)	0.141
**CC vs CT**							
Total	77	Random	125.60	0.000	39.5%	0.972(0.928 1.019)	0.241
Caucasian	38	Random	82.78	0.000	55.3%	0.961(0.923 1.002)	0.060
Asian	27	Fixed	21.48	0.717	0.0%	1.026 (0.958 1.098)	0.462
Mixed population	8	Fixed	2.59	0.920	0.0%	0.975 (0.901 1.055)	0.531
African	4	Random	14.13	0.003	78.8%	0.710 (0.498 1.010)	0.057
**CT vs TT**							
Total	77	Random	123.62	0.000	38.5%	1.113(1.037 1.195)	**0.003**
Caucasian	38	Random	76.06	0.000	51.4%	1.102(1.032 1.177)	**0.004**
Asian	27	Random	40.75	0.033	36.2%	1.180(1.079 1.291)	**0.000**
Mixed population	8	Fixed	4.87	0.676	0.0%	1.142(1.005 1.296)	**0.041**
African	4	Fixed	0.41	0.938	0.0%	1.089(0.476 2.494)	0.840
**CC vs CT+TT**							
Total	77	Random	153.54	0.000	50.5%	0.994(0.946 1.044)	0.806
Caucasian	38	Random	88.16	0.000	58.0%	0.982(0.944 1.021)	0.356
Asian	27	Fixed	37.63	0.065	30.9%	1.069(1.003 1.140)	**0.041**
Mixed population	8	Fixed	4.34	0.740	0.0%	1.002(0.930 1.080)	0.960
African	4	Random	16.22	0.001	81.5%	0.714(0.508 1.002)	0.051
**CC+CT vs TT**							
Total	77	Random	148.27	0.000	48.7%	1.097(1.019 1.182)	**0.015**
Caucasian	38	Random	79.69	0.000	53.6%	1.086(1.021 1.156)	**0.009**
Asian	27	Random	54.62	0.001	52.4%	1.198(1.101 1.303)	**0.000**
Mixed population	8	Fixed	6.75	0.455	0.0%	1.126(0.999 1.270)	0.052
African	4	Fixed	2.85	0.415	0.0%	1.123(1.073 1.177)	0.469
**C-allele vs T-allele**							
Total	77	Random	194.58	0.000	60.9%	1.014(0.973 1.056)	0.508
Caucasian	38	Random	96.87	0.000	61.8%	1.000(0.948 1.056)	0.986
Asian	27	Random	63.92	0.000	59.3%	1.054 (0.974 1.140)	0.193
Mixed population	8	Fixed	7.09	0.420	1.2%	1.028(0.970 1.088)	0.351
African	4	Random	14.68	0.002	79.6%	0.664(0.315 1.400)	0.282

**Note:** There are 71 studies which some one includes two or three researches in one work, including 77 case-control or cohort researches.

In the stratified analysis by ethnicity, significantly increased risks were found among Caucasians for *CC vs TT* (OR = 1.076; 95%CI = 1.008–1.150; *I^2^ = *52.3%, **Figure** **S3**), *CT vs TT* (OR = 1.102; 95%CI = 1.032–1.177; *I^2^ = *51.4%, **Figure** **S4**) and dominant model (OR = 1.086; 95%CI = 1.021 −1.156; *I^2^ = *53.6%, **Figure** **S5**), (**Table** **1**). Simultaneously, significantly increased risks were also found among Asians for *CC vs TT* (OR  = 1.226; 95%CI  = 1.116–1.346; *I^2^  = *55.3%, **Figure** **S3**), *CT vs TT* (OR  = 1.180; 95%CI  = 1.079–1.291; *I^2^  = *36.2%, **Figure** **S4**), recessive (OR  = 1.069; 95%CI  = 1.003–1.140; *I^2^  = *30.9%, **Figure** **S6**) and dominant model (OR  = 1.198; 95%CI  = 1.101–1.303; *I^2^  = *52.4%, **Figure** **S5**), (**Table** **1**). For Mixed populations, significantly increased risks were found for *CT vs TT* (OR  = 1.142; 95%CI  = 1.005–1.296; *I^2^  = *0.0%, **Figure** **S4**) (**Table** **1**). However, no significant associations were found in Africans for all genetic models **(** **Table** **1** **).**

There was significant heterogeneity for all genetic model comparisons among worldwide populations. After assessing the source of heterogeneity for all genetic model comparison by subgroup analysis on ethnicity the heterogeneity was partly decreased or removed. However, there was still significant heterogeneity among different descent population studies. When we deleted the study on evaluating different descent populations for departure from *HWE*, the heterogeneity was completely removed.

### Sensitivity analysis

In order to compare the difference and evaluate the sensitivity of the meta-analyses, we conducted one-way sensitivity analysis to evaluate the stability of the meta-analysis. The statistical significance of the results was not altered when any single study was omitted, confirming the stability of the results. Hence, results of the sensitivity analysis suggest that the data in this meta-analysis are relatively stable and credible (Date was not shown).

### Publication bias

**Figure 4:**
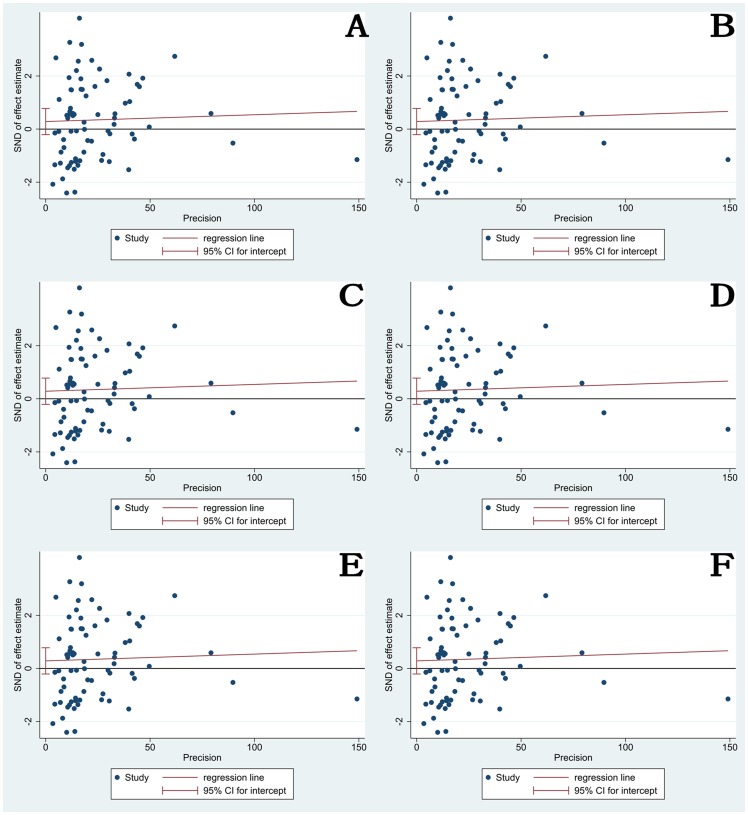
Eegger's funnel plot with pseudo 95% confidence limit under all different genetic models of 677C>T genotype(A: *CC vs TT*; B: *CC* vs *CT*; C: *CT* vs *TT*; D: *CC vs CT+TT*; E: *CC+CT vs TT*; F: *C-allele vs T-allele*).

Begg's funnel plot and Egger's test were performed to assess the publication bias of the literature. The shapes of the funnel plots did not reveal any evidence of obvious asymmetry in all comparison models. Furthermore, Egger's test was used to provide statistical evidence for funnel plot symmetry (**Figure** **4**). The results still did not suggest any evidence of publication bias.

## Discussion

It is well recognized that there is a range of individual susceptibility to the CRC even with identical environmental exposure. Single nucleotide polymorphism (SNP) is the most common form of human genetic variation, and may contribute to individual's susceptibility to cancer, however, the underlying molecular mechanism is unknown. Therefore, genetic susceptibility to cancer has been a research focus in scientific community. Some variants, especially those in the promoter regions of genes, may affect either the expression or activity levels of enzymes [90]–[93] and therefore may be mechanistically associated with cancer risk. Previous studies investigating the association between *MTHFR C677T* polymorphism and CRC risk have provided inconsistent results. These inconsistent results are possibly because most of those studies involved no more than a few hundred CRC cases, which is too few to assess any genetic effects reliably, which resulted in a small effect of the polymorphism on CRC risk and relatively low statistical power of the published studies. Hence, a meta-analysis was needed to provide a quantitative approach for combining the results of various studies with the same topic, and for estimating and explaining their diversity.

Meta-analysis has been considered as an important tool to more precisely define the effect of selected genetic polymorphisms on risk of disease and to identify potentially important sources of between-study heterogeneity. Previous meta-analysis included only 8 case-control studies in the analysis of Asian population, which was too little to confirm the association between *MTHFR C677T* polymorphism and CRC risk [94]. Yang et al included 21 studies to research the associations in his work but only from Asian descent [95]. In order to provide the most comprehensive assessment of the association between *MTHFR C677T* polymorphism and CRC risk in worldwide populations, we did an updated meta-analysis of all available studies. At last, we performed this updated meta-analysis on the association between *MTHFR C677T* polymorphism and CRC risk by critically reviewing 71 individual studies including 31,572 cases and 44,066 controls.

In the present meta-analysis, we found that the variant genotypes of the *MTHFR C677T* polymorphisms were significantly associated with CRC risk. Simultaneously, subgroup analyses by ethnicity further identified this association. We found that the variant genotype of the *MTHFR C677T* polymorphism, in Caucasian population, was associated with significant increase in CRC risk. The same results were detected among Asian, and Mixed populations. Although the *MTHFR C677T* polymorphism may be associated with DNA repair activity, no significant associations of some variant genotypes with CRC risk were found in Caucasian, Asian and Mixed populations, suggesting the influence of the genetic variant may be masked by the presence of other as-yet unidentified causal genes involved in CRC. Moreover, there is no significant association was found among African descent populations.

Possible limitations of this meta-analysis should be acknowledged. Firstly, publication bias may have occurred because only published researches were included in this study. Though Funnel plots were performed to access the publication bias in this meta-analysis and the outcome suggested that publication bias was not evident in the present study, but the publication bias in the present analysis was still not negligible. Secondly, misclassification bias was possible. For example, most studies could not exclude latent cancer cases in the control group. The controls in some studies were selected from non-cancer patients, while the controls in other several studies were just selected from asymptomatic individuals. Thirdly, in the subgroup analyses by ethnicity, only four studies in African populations included in our meta-analysis, which means relatively limited study number made it impossible to perform ethnic subgroup analysis. Thus, additional studies are warranted to evaluate the effect of this functional polymorphism on CRC risk in different ethnicities, especially in African descent populations. Finally, gene-environmental interactions were not fully addressed in this work for the lack of sufficient data. As we know, aside from genetic factor, smoking, diet habit, smoking, drinking status and some environmental risk factors are major risk factors for CRC. However we didn't perform subgroup analyses based on environmental explosion owing to the limited reported information on such associations in the included studies. In addition, our analysis did not consider the possibility of gene-gene or SNP-SNP interactions or the possibility of linkage disequilibrium between polymorphisms. Considering that, a more precise analysis should be conducted adjusted by all the factors stated above.

Despite of those limitations, this meta-analysis provided evidence of the association between the *MTHFR C677T* polymorphisms and CRC risk, supporting the hypothesis that *MTHFR C677T* polymorphism is contributed to overall CRC risk. In subgroup analysis, the same results were found in Caucasian, Asian and Mixed populations, but not in African descent men. In order to verify our findings, well-designed studies are needed to further evaluate the association between the *MTHFR C677T* polymorphism and CRC risk.

## Supporting Information

Figure S1 **Forest plot of colorectal cancer susceptibility associated with** ***MTHFR 677C>T*** **polymorphism (for** ***CT*** **vs** ***TT*** **).**
(TIF)

Figure S2 **Forest plot of colorectal cancer susceptibility associated with** ***MTHFR 677C>T*** **polymorphism at additive model (** ***C-allele vs T-allele*** **).**
(TIF)

Figure S3 **Forest plot of colorectal cancer susceptibility associated with** ***MTHFR 677C>T*** **polymorphism in different descent populations (** ***CC*** **vs** ***TT*** **).**
(TIF)

Figure S4 **Forest plot of colorectal cancer susceptibility associated with** ***MTHFR 677C>T*** **polymorphism in different descent populations (** ***CT*** **vs** ***TT*** **).**
(TIF)

Figure S5 **Forest plot of colorectal cancer susceptibility associated with** ***MTHFR 677C>T*** **polymorphism in different descent populations at dominant model (** ***CC + CT*** **vs** ***TT*** **).**
(TIF)

Figure S6 **Forest plot of colorectal cancer susceptibility associated with** ***MTHFR 677C>T*** **polymorphism in different descent populations at recessive model (** ***CC*** **vs** ***CT + TT*** **).**
(TIF)

Table S1 **Main characters of studies included in this meta-analysis.**
(DOC)
