# Supplementary figures and images for: The 677C>T (rs1801133) Polymorphism in the MTHFR Gene Contributes to Colorectal Cancer Risk: A Meta-Analysis Based on 71 Research Studies
Source: PLoS One. 2013 Feb 20;8(2):e55332. doi: 10.1371/journal.pone.0055332 (PMC3577825; doi:10.1371/journal.pone.0055332)

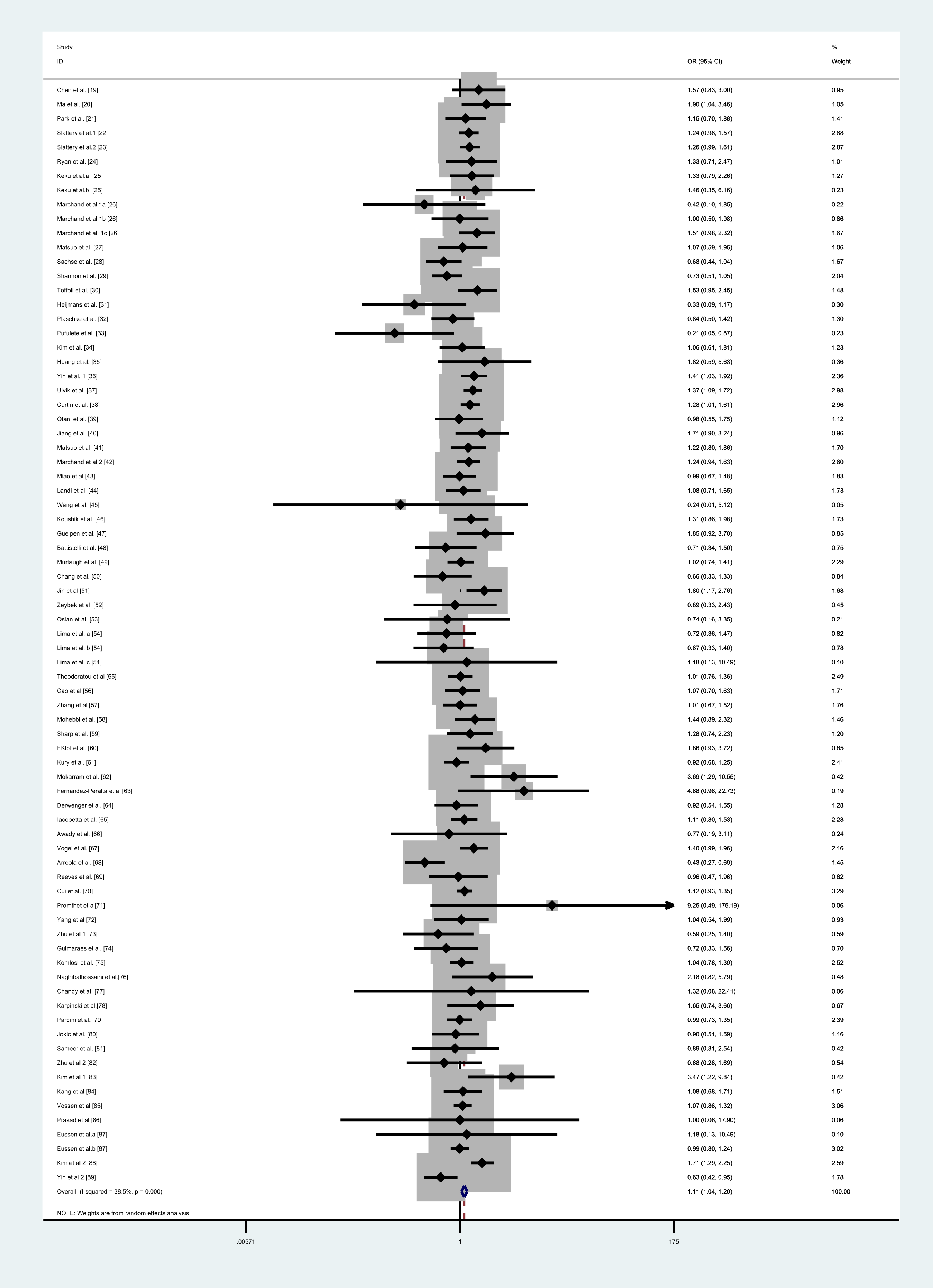

Supplement: Figure S1 — Forest plot of colorectal cancer susceptibility associated with MTHFR 677C>T polymorphism (for CT vs TT ). (TIF) [file pone.0055332.s001.tif]

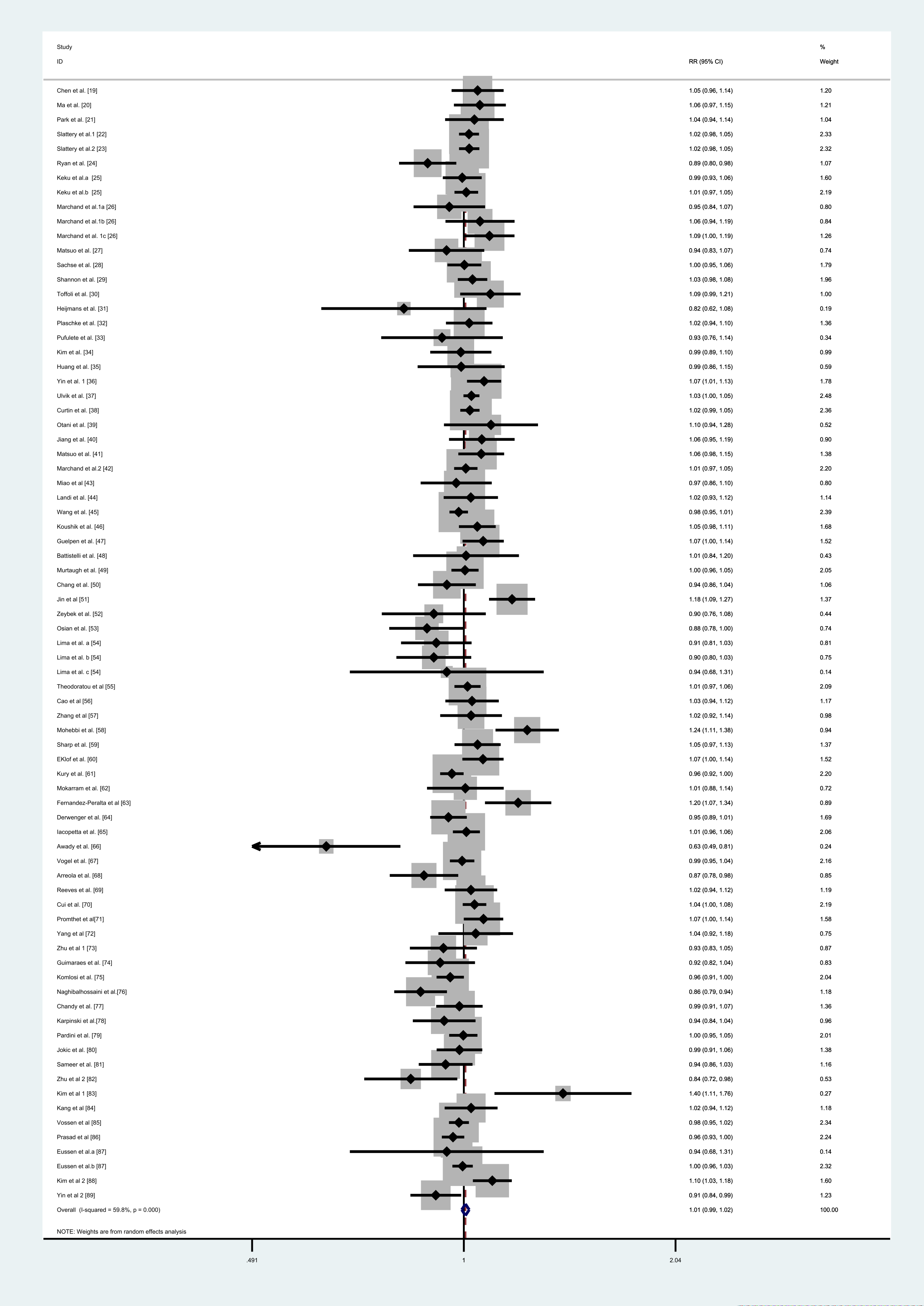

Supplement: Figure S2 — Forest plot of colorectal cancer susceptibility associated with MTHFR 677C>T polymorphism at additive model ( C-allele vs T-allele ). (TIF) [file pone.0055332.s002.tif]

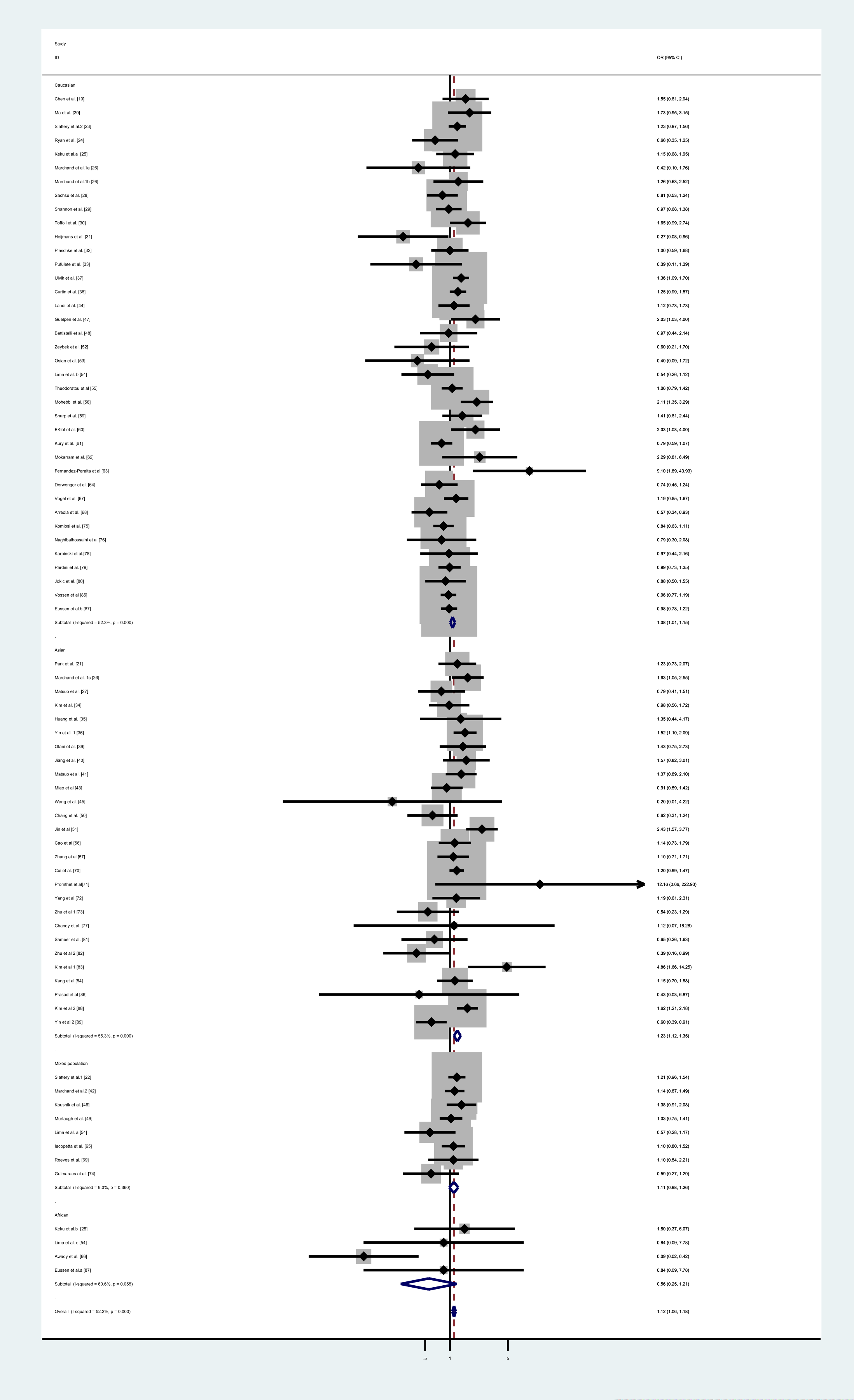

Supplement: Figure S3 — Forest plot of colorectal cancer susceptibility associated with MTHFR 677C>T polymorphism in different descent populations ( CC vs TT ). (TIF) [file pone.0055332.s003.tif]

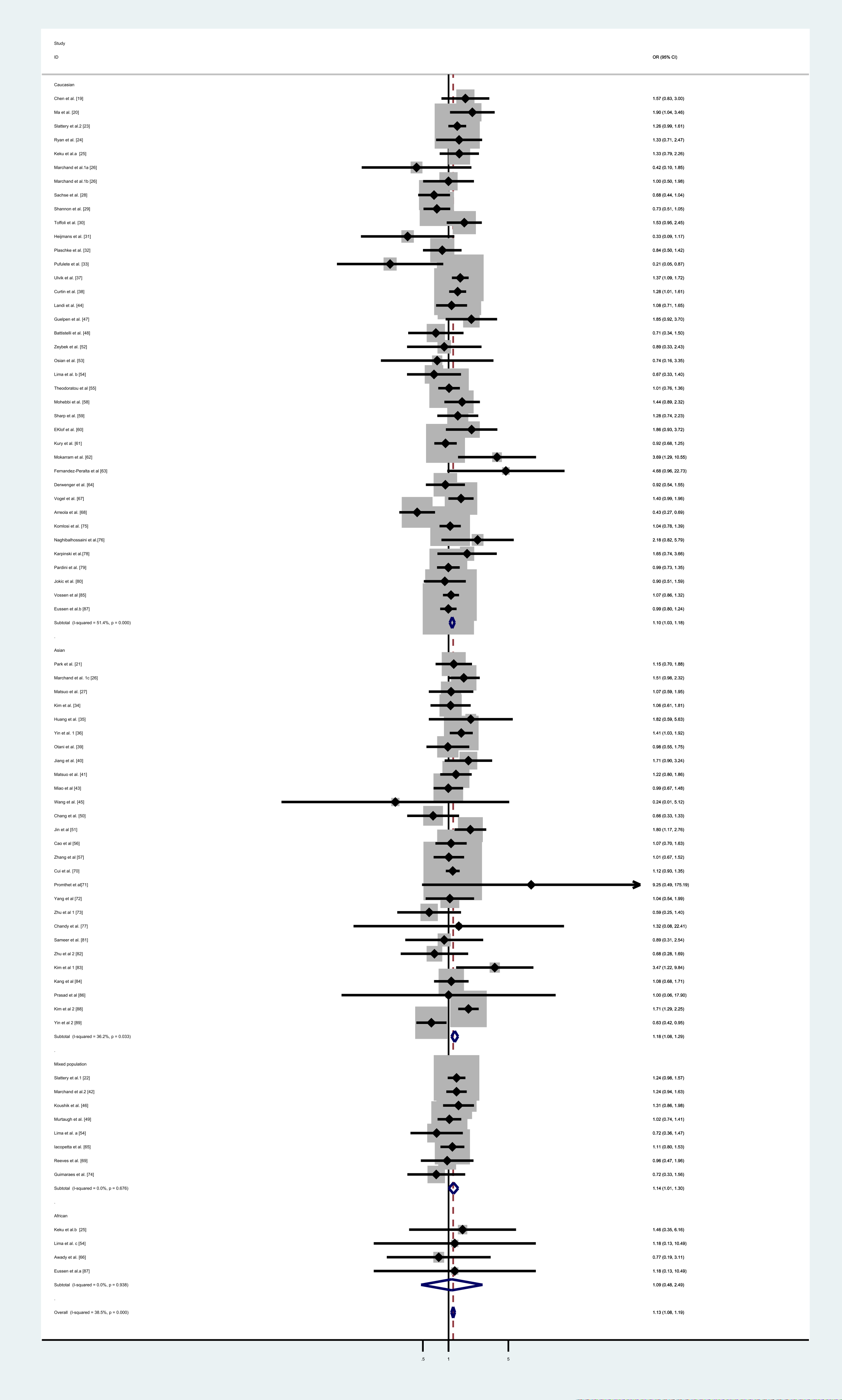

Supplement: Figure S4 — Forest plot of colorectal cancer susceptibility associated with MTHFR 677C>T polymorphism in different descent populations ( CT vs TT ). (TIF) [file pone.0055332.s004.tif]

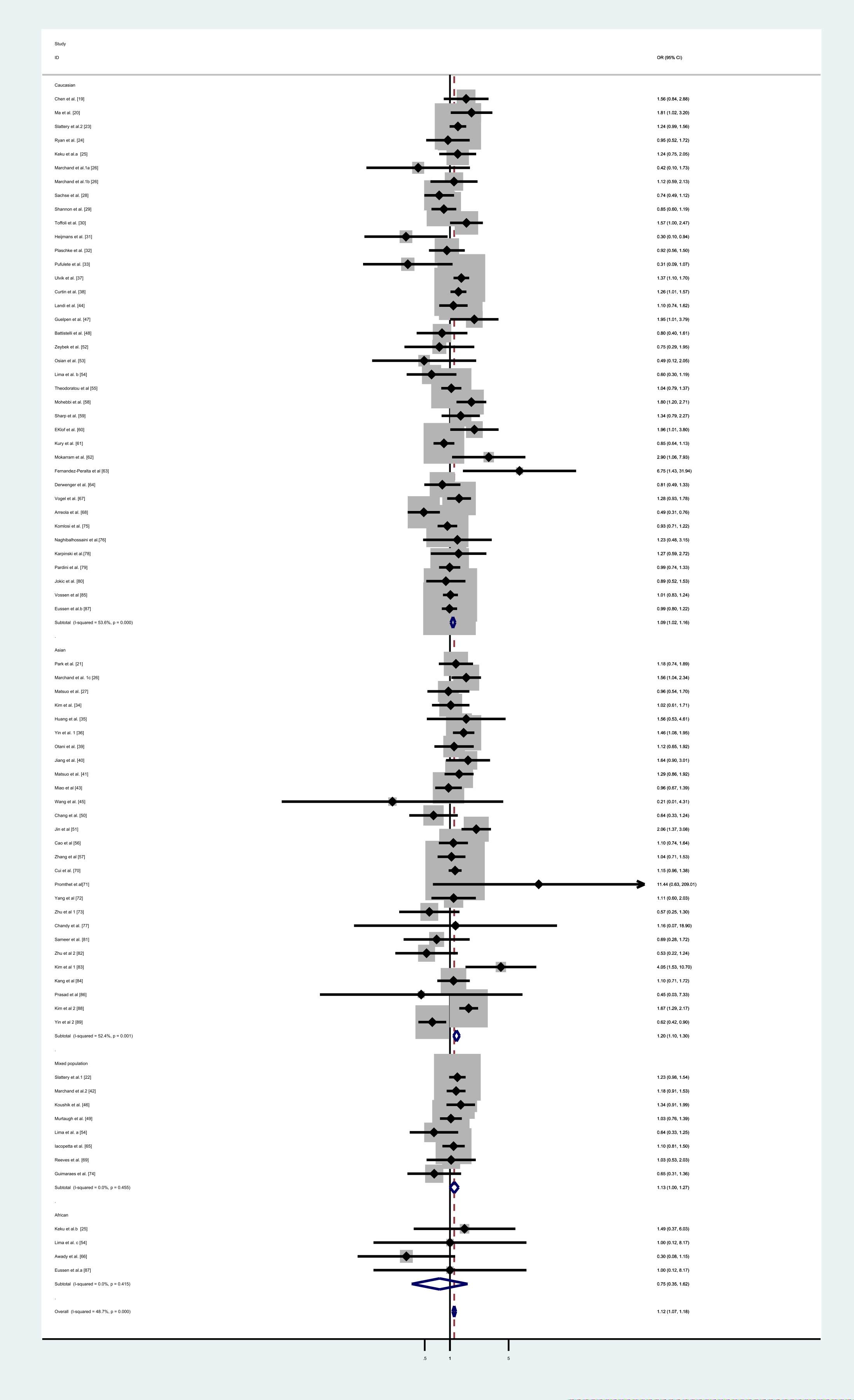

Supplement: Figure S5 — Forest plot of colorectal cancer susceptibility associated with MTHFR 677C>T polymorphism in different descent populations at dominant model ( CC + CT vs TT ). (TIF) [file pone.0055332.s005.tif]

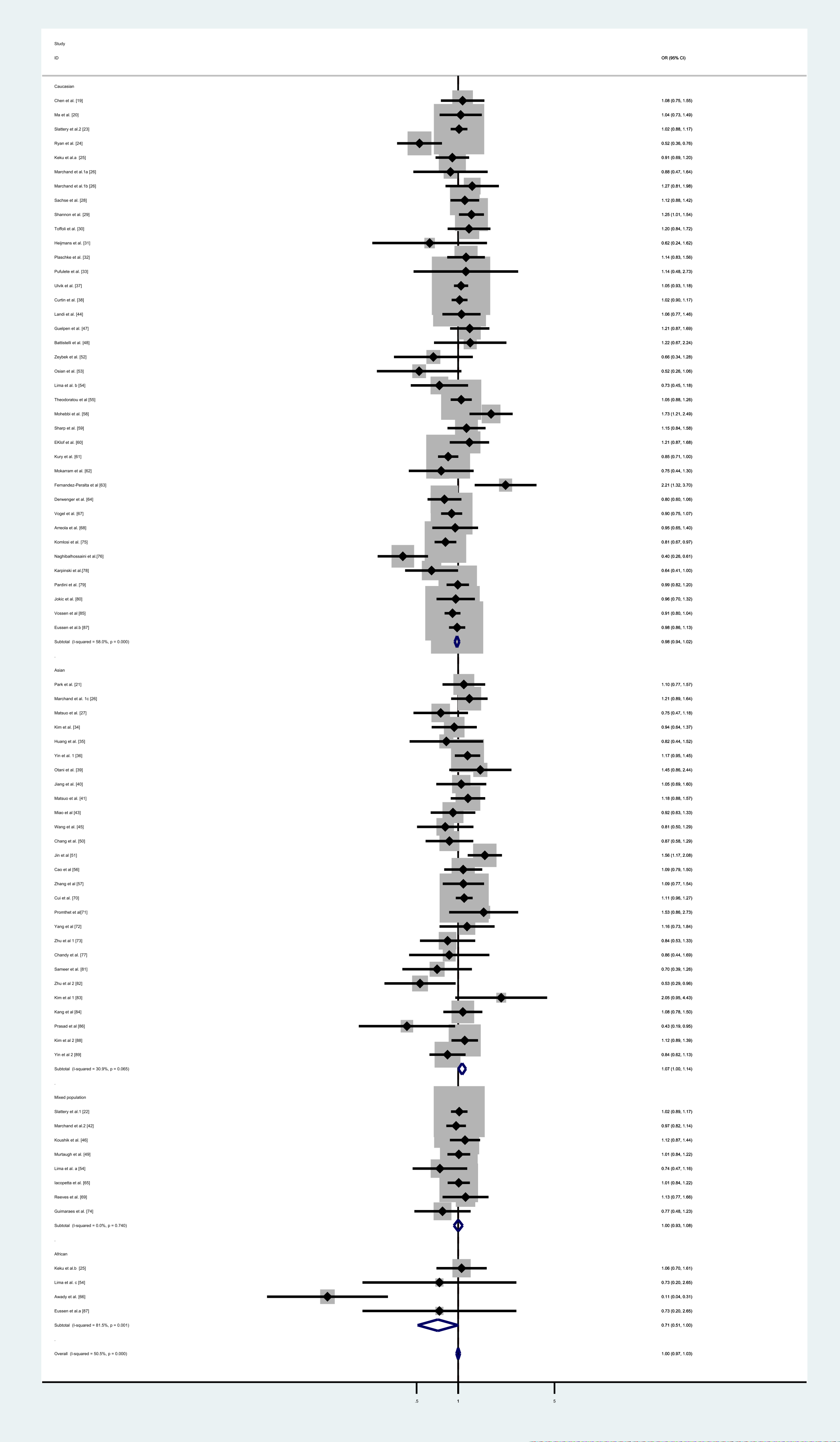

Supplement: Figure S6 — Forest plot of colorectal cancer susceptibility associated with MTHFR 677C>T polymorphism in different descent populations at recessive model ( CC vs CT + TT ). (TIF) [file pone.0055332.s006.tif]
