# Supplementary material for: The 677C>T (rs1801133) Polymorphism in the MTHFR Gene Contributes to Colorectal Cancer Risk: A Meta-Analysis Based on 71 Research Studies
Source: PLoS One. 2013 Feb 20;8(2):e55332. doi: 10.1371/journal.pone.0055332 (PMC3577825; doi:10.1371/journal.pone.0055332)
Supplement: Table S1 — Main characters of studies included in this meta-analysis. (DOC) [file pone.0055332.s007.doc]

Table S1. Main characters of studies included in this meta-analysis.

| **First author**  **[Inference]** | **Year** | **Ethnicity** | **Cases** | | | **Controls** | | | ***P* for *HWE*** |
| --- | --- | --- | --- | --- | --- | --- | --- | --- | --- |
|  |  |  | CC | CT | TT | CC | CT | TT |  |
| Chen et al. [19] | 1996 | Caucasian | 67 | 64 | 13 | 280 | 263 | 84 | 0.08 |
| Ma et al. [20] | 1997 | Caucasian | 92 | 92 | 18 | 145 | 132 | 49 | **0.04** |
| Park et al. [21] | 1999 | Asian | 65 | 107 | 28 | 140 | 246 | 74 | **0.05** |
| Slattery et al.^1^ [22] | 1999 | Mixed population | 673 | 655 | 139 | 827 | 787 | 207 | 0.34 |
| Slattery et al. ^2^ [23] | 2000 | Caucasian | 654 | 641 | 137 | 802 | 763 | 206 | 0.23 |
| Ryan et al. [24] | 2001 | Caucasian | 49 | 73 | 14 | 439 | 326 | 83 | **0.05** |
| Keku et al.^a^ [25] | 2002 | Caucasian | 144 | 140 | 24 | 265 | 223 | 51 | 0.68 |
| Keku et al.^b^ [25] | 2002 | African | 198 | 43 | 3 | 264 | 59 | 6 | 0.21 |
| Marchand et al. ^1a^ [26] | 2002 | Caucasian | 44 | 27 | 6 | 53 | 32 | 3 | 0.49 |
| Marchand et al. ^1b^[26] | 2002 | Caucasian | 66 | 64 | 19 | 66 | 81 | 24 | 0.91 |
| Marchand et al. ^1c^ [26] | 2002 | Asian | 126 | 153 | 43 | 138 | 182 | 77 | 0.22 |
| Matsuo et al. [27] | 2002 | Asian | 39 | 81 | 22 | 81 | 124 | 36 | 0.30 |
| Sachse et al. [28] | 2002 | Caucasian | 238 | 199 | 53 | 271 | 272 | 49 | 0.09 |
| Shannon et al. [29] | 2002 | Caucasian | 249 | 197 | 55 | 533 | 560 | 114 | 0.06 |
| Toffoli et al. [30] | 2003 | Caucasian | 93 | 145 | 38 | 83 | 140 | 56 | 0.83 |
| Heijmans et al. [31] | 2003 | Caucasian | 7 | 7 | 4 | 392 | 322 | 61 | 0.81 |
| Plaschke et al. [32] | 2003 | Caucasian | 133 | 120 | 34 | 149 | 159 | 38 | 0.65 |
| Pufulete et al. [33] | 2003 | Caucasian | 16 | 6 | 6 | 41 | 29 | 6 | 0.78 |
| Kim et al. [34] | 2004 | Asian | 86 | 122 | 35 | 83 | 109 | 33 | 0.77 |
| Huang et al. [35] | 2003 | Asian | 36 | 40 | 6 | 40 | 33 | 9 | 0.58 |
| Yin et al. ^1^ [36] | 2004 | Asian | 270 | 330 | 85 | 278 | 367 | 133 | 0.53 |
| Ulvik et al. [37] | 2004 | Caucasian | 1103 | 899 | 157 | 1092 | 886 | 212 | 0.10 |
| Curtin et al. [38] | 2004 | Caucasian | 729 | 717 | 149 | 886 | 852 | 226 | 0.36 |
| Otani et al. [39] | 2005 | Asian | 32 | 49 | 25 | 51 | 114 | 57 | 0.68 |
| Jiang et al. [40] | 2005 | Asian | 51 | 59 | 15 | 134 | 143 | 62 | **0.03** |
| Matsuo et al. [41] | 2005 | Asian | 106 | 114 | 36 | 289 | 348 | 134 | 0.10 |
| Marchand et al.^2^ [42] | 2005 | Mixed population | 384 | 329 | 87 | 987 | 779 | 255 | **0.00** |
| Miao et al [43] | 2005 | Asian | 53 | 87 | 58 | 133 | 201 | 133 | **0.00** |
| Landi et al. [44] | 2005 | Caucasian | 128 | 158 | 64 | 109 | 139 | 61 | 0.17 |
| Wang et al. [45] | 2006 | Asian | 257 | 43 | 2 | 255 | 36 | 0 | 0.26 |
| Koushik et al. [46] | 2006 | Mixed population | 166 | 145 | 38 | 355 | 327 | 112 | **0.01** |
| Guelpen et al. [47] | 2006 | Caucasian | 123 | 85 | 12 | 212 | 161 | 42 | 0.17 |
| Battistelli et al. [48] | 2006 | Caucasian | 32 | 40 | 21 | 30 | 51 | 19 | 0.75 |
| Murtaugh et al. [49] | 2007 | Mixed population | 362 | 305 | 84 | 469 | 398 | 112 | **0.02** |
| Chang et al. [50] | 2007 | Asian | 85 | 86 | 24 | 92 | 87 | 16 | 0.47 |
| Jin et al [51] | 2007 | Asian | 143 | 154 | 37 | 162 | 236 | 102 | 0.35 |
| Zeybek et al. [52] | 2007 | Caucasian | 18 | 27 | 7 | 64 | 65 | 15 | 0.80 |
| Osian et al. [53] | 2007 | Caucasian | 38 | 25 | 6 | 47 | 17 | 3 | 0.38 |
| Lima et al. ^a^ [54] | 2007 | Mixed population | 41 | 46 | 15 | 143 | 127 | 30 | 0.82 |
| Lima et al. ^b^ [54] | 2007 | Caucasian | 36 | 40 | 14 | 143 | 127 | 30 | 0.82 |
| Lima et al. ^c^ [54] | 2007 | African | 4 | 5 | 1 | 143 | 127 | 30 | 0.82 |
| Theodoratou et al [55] | 2008 | Caucasian | 447 | 441 | 111 | 439 | 455 | 116 | 0.60 |
| Cao et al [56] | 2008 | Asian | 109 | 154 | 52 | 121 | 183 | 66 | 0.82 |
| Zhang et al [57] | 2008 | Asian | 97 | 136 | 67 | 91 | 139 | 69 | 0.26 |
| Mohebbi et al. [58] | 2008 | Caucasian | 117 | 68 | 49 | 94 | 80 | 83 | **0.00** |
| Sharp et al. [59] | 2008 | Caucasian | 117 | 111 | 23 | 170 | 177 | 47 | 1.53 |
| EKlof et al. [60] | 2008 | Caucasian | 123 | 85 | 12 | 212 | 160 | 42 | 0.15 |
| Kury et al. [61] | 2008 | Caucasian | 484 | 432 | 107 | 577 | 443 | 101 | 0.23 |
| Mokarram et al. [62] | 2008 | Caucasian | 64 | 80 | 7 | 40 | 31 | 10 | 0.31 |
| Fernandez-Peralta et al [63] | 2009 | Caucasian | 89 | 52 | 2 | 44 | 50 | 9 | 0.32 |
| Derwenger et al. [64] | 2009 | Caucasian | 273 | 216 | 55 | 167 | 107 | 25 | 0.19 |
| Iacopetta et al. [65] | 2009 | Mixed population | 382 | 386 | 82 | 428 | 429 | 101 | 0.67 |
| Awady et al. [66] | 2009 | African | 6 | 23 | 6 | 44 | 20 | 4 | 0.41 |
| Vogel et al. [67] | 2009 | Caucasian | 318 | 320 | 51 | 876 | 750 | 167 | 0.72 |
| Arreola et al. [68] | 2009 | Caucasian | 124 | 126 | 119 | 59 | 79 | 32 | 0.54 |
| Reeves et al. [69] | 2009 | Mixed population | 105 | 83 | 18 | 101 | 91 | 19 | 0.82 |
| Cui et al. [70] | 2010 | Asian | 622 | 923 | 284 | 540 | 863 | 297 | 0.13 |
| Promthet et al[71] | 2010 | Asian | 104 | 26 | 0 | 94 | 31 | 5 | 0.24 |
| Yang et al [72] | 2010 | Asian | 58 | 61 | 22 | 62 | 75 | 28 | 0.52 |
| Zhu et al ^1^ [73] | 2010 | Asian | 88 | 102 | 26 | 50 | 53 | 8 | 0.23 |
| Guimaraes et al. [74] | 2010 | Mixed population | 48 | 50 | 15 | 92 | 79 | 17 | 0.99 |
| Komlosi et al. [75] | 2010 | Caucasian | 398 | 427 | 126 | 442 | 380 | 117 | **0.01** |
| Naghibalhossaini et al.  [76] | 2010 | Caucasian | 64 | 80 | 7 | 150 | 68 | 13 | 0.16 |
| Chandy et al. [77] | 2010 | Asian | 74 | 25 | 1 | 66 | 19 | 1 | 0.78 |
| Karpinski et al.[78] | 2010 | Caucasian | 74 | 97 | 15 | 71 | 55 | 14 | 0.49 |
| Pardini et al. [79] | 2010 | Caucasian | 281 | 309 | 76 | 583 | 638 | 156 | 0.18 |
| Jokic et al. [80] | 2011 | Caucasian | 139 | 130 | 31 | 142 | 130 | 28 | 0.82 |
| Sameer et al. [81] | 2011 | Asian | 59 | 18 | 9 | 121 | 27 | 12 | **0.00** |
| Zhu et al ^2^ [82] | 2011 | Asian | 29 | 42 | 15 | 49 | 41 | 10 | 0.74 |
| Kim et al ^1^ [83] | 2011 | Asian | 30 | 30 | 7 | 15 | 21 | 17 | 0.13 |
| Kang et al [84] | 2011 | Asian | 87 | 134 | 34 | 145 | 238 | 65 | **0.04** |
| Vossen et al [85] | 2011 | Caucasian | 737 | 832 | 202 | 795 | 807 | 209 | 0.85 |
| Prasad et al [86] | 2011 | Asian | 97 | 12 | 1 | 228 | 12 | 1 | 3.82 |
| Eussen et al.^a^ [87] | 2011 | African | 4 | 5 | 1 | 143 | 127 | 30 | 0.82 |
| Eussen et al.^b^ [87] | 2011 | Caucasian | 567 | 608 | 154 | 1019 | 1076 | 271 | 0.61 |
| Kim et al ^2^ [88] | 2012 | Asian | 265 | 393 | 129 | 205 | 289 | 162 | **0.00** |
| Yin et al ^2^ [89] | 2012 | Asian | 124 | 167 | 79 | 139 | 178 | 53 | 0.74 |
| ^a,b,c^ They are different case-control studies in one work; *HWE:* Hardy–Weinberg Equilibrium. | | | | | | | | | |
